# Bevacizumab for non-small cell lung cancer patients with brain metastasis: A meta-analysis

**DOI:** 10.1515/med-2020-0192

**Published:** 2020-07-01

**Authors:** Ping Liang, Yu-Dong Wang, Zong-Min Wei, Qi-Jun Deng, Tong Xu, Jiang Liu, Na Luo, Juan Hou

**Affiliations:** Department of Pharmacy, The Fourth Hospital of Hebei Medical University, No. 169, Tianshan Street, Shijiazhuang, Hebei 050011, China; Department of Oncology, Hebei Medical University, Shijiazhuang, Hebei 050011, China; Department of Science and Technology, Beijing You’an Hospital, Capital Medical University, Beijing 100069, China

**Keywords:** bevacizumab, NSCLC, brain metastasis, efficacy, safety

## Abstract

This study evaluates the efficacy and safety of bevacizumab (BEV) in the treatment of non-small cell lung cancer (NSCLC) patients with brain metastases (BM) by performing meta-analyses of response and survival indices. Seventeen studies were included. BEV treatment was associated with a lower new BM incidence (hazard ratio: 0.30 [95% confidence interval (CI): 0.14, 0.46]) during follow-up. Disease control rate (DCR) of BEV-treated patients with BM was 91% [95% CI: 85, 95]. However, intracranial DCR was relatively higher (94% [95% CI: 87, 98]) than extracranial DCR (86% [95% CI: 74, 96]). DCR of NSCLC patients with BM was significantly better with BEV than with control therapies (odds ratio: 2.71 [95% CI: 1.26, 5.86], *P* = 0.01). Progression-free survival (PFS) of BEV-treated patients with and without BM was 7.1 months [95% CI: 6.2, 8.0] and 7.4 months [95% CI: 6.3, 8.4], respectively. Intracranial PFS of BEV-treated patients with BM was 8.0 months [95% CI: 6.0, 10.0]. Overall survival of BEV-treated NSCLC patients with and without BM was 13.5 months [95% CI: 11.4, 15.6] and 12.5 months [95% CI: 10.2, 14.8], respectively. The incidence of bleeding/hemorrhage in the central nervous system was 1% with BEV treatment.

## 1 Introduction

Non-small cell lung cancer (NSCLC) comprises 80–85% of all types of lung cancer and is a leading cause of cancer-related mortality among men and women worldwide [1]. Of all NSCLC cases, 60–70% are diagnosed with stage IIIB or IV for which the prognosis is poor [2]. Active or passive smoking, alcohol use, air pollution, occupational exposure, and cancer susceptibility genes are important risk factors for NSCLC [3]. The 5-year survival of NSCLC patients after diagnosis is only 15% [3]. Age, stage of cancer, performance status, mediastinal lymph node status at diagnosis, comorbidity, leukocyte and neutrophil count, delay of management, and antitumor treatment are important determinants of the prognosis [4,5]. Brain metastasis (BM) is a common consequence of lung cancer, with an incidence of approximately 10% at initial diagnosis and up to 40% during the progression of disease [6,7].

Conventionally, chemotherapy and radiotherapy are the mainstays of treatment for BM. The median progression-free survival (PFS) of NSCLC patients with BM is approximately 8–9 months after stereotactic radiosurgery or whole-brain radiation therapy and chemotherapy [8,9]. With the development of targeted therapies, clinical and survival outcomes of NSCLC patients have been improved. Among tyrosine kinase inhibitors (TKIs), osimertinib as first-line treatment significantly improves PFS compared with first-generation TKIs (19 versus 10 months) in NSCLC patients [10]. Although patients can benefit from such therapies, some limitations still exist such as drug resistance to epidermal growth factor receptor and anaplastic lymphoma kinase inhibitors, the impaired ability of drugs to cross the blood–brain barrier, and impaired neurocognitive function caused by the whole-brain radiation therapy [11]. Therefore, advanced treatment options are needed for NSCLC patients.

Bevacizumab (BEV), a recombinant humanized monoclonal antibody against vascular endothelial growth factor (VEGF), has exhibited efficacy in several cancer types. This antibody can inhibit tumor growth by competing with VEGF for VEGF receptors. Several trials are conducted to evaluate the efficacy and safety of BEV, which are also well reviewed. A meta-analysis of 24 randomized controlled trials found that BEV treatment improved the overall survival (OS) and PFS in patients with metastatic solid tumors but was associated with a statistically higher incidence of fatal adverse events (AEs) overall and an increased risk of fatal pulmonary hemorrhage in patients with lung cancer [12]. However, a meta-analysis of cancer patients with BM found that BEV treatment was not associated with a significantly increased risk of intracerebral hemorrhage [13].

The meta-analyses of patients with advanced NSCLC have also shown that BEV treatment with chemotherapy was able to significantly prolong OS and PFS and was well tolerated [14,15]. However, clinical potentials of BEV for NSCLC patients with BM are not clear. The aim of this study was to systematically review the studies that have evaluated the efficacy and safety of BEV in NSCLC patients with BM to perform meta-analyses of important indices to gain a refined evidence of the efficacy and safety of BEV in the treatment of NSCLC patients with BM.

## 2 Methods

### 2.1 Search strategy

Electronic databases (Cochrane Library, Clinical Trials, CNKI, Embase, Google Scholar, Ovid SP, and PubMed) were searched for relevant articles published before January 2020. The following terms were used for the search: bevacizumab, Avastin, non-small cell lung cancer, brain, central nervous system, CNS, metastases, efficacy, safety, response, and survival. After identifying the relevant articles, we also checked the reference lists of the included studies to find additional relevant studies.

### 2.2 Eligibility criteria

Inclusion criteria were that the study (1) recruited adult NSCLC patients (tissue or cell diagnosed) with BM (diagnosed with computed tomography or magnetic resonance imaging) who were treated with BEV alone or in combination with other therapies; (2) recruited adult NSCLC patients without BM and treated them with BEV to examine the incidence of BM during study period; and (3) reported quantitative data of efficacy and safety, especially the disease control rate (DCR), the overall response rate (ORR), PFS, OS, and the incidence of AEs. Exclusion criteria were that the study: (1) did not differentiate the outcomes by BM, (2) used BEV for brain lesions other than BM from NSCLC, (3) had a follow-up completion rate of less than 60%, and (4) published as case reports.

### 2.3 Data extraction and study quality assessment

The following data were extracted from research articles of the qualified studies: study design and conduct variables; demographic information and clinical and pathological characteristics of the patients; performance status and mutational characteristics of the patients; treatments and dosage schedules; treatment history; outcome data, especially for PFS, OS, DCR, ORR, treatment-related mortality, grade 3 or 4 AEs; and associational data. Three reviewers identified the eligible articles independently by the following eligibility criteria. Any disagreement was resolved with mutual discussions or by involving a senior colleague. Data extraction was also carried out by 3 reviewers independently and then outputs were unified by arriving at consensus when any disagreement arose. Study quality assessment was performed with the Newcastle-Ottawa Scale for observational studies.

### 2.4 Statistical analyses

The hazard ratios (HRs) reported by the individual studies were pooled under random effects model to achieve HR estimates of (1) the survival of BEV-treated versus control NSCLC patients with BM, (2) the survival of BEV-treated NSCLC patients with BM versus without BM, and (3) the incidence versus no incidence of BM during the study in patients who were without BM at study start.

A meta-analysis of the odds ratios (ORs) was performed to determine the significance of difference in the DCR and ORR between the BEV and control treatments in the NSCLC patients with BM using binomial data reported by the individual studies. In a separate meta-analysis, the median PFS and OS rates reported by the individual studies were pooled under random effects model by subgrouping the patients with and without BM.

The meta-analyses of the proportions with Freeman–Tukey double arcsine transformation were performed to achieve the complete remission, partial remission, stable disease, progressive disease, ORR, DCR, 1-year OS, and the incidence of bleeding or hemorrhage in the central nervous system (CNS) rates by subgrouping the outcomes with regard to intracranial and extracranial or BM and no BM outcomes. The clinical significance of the outcomes was decided based on the overall/subgroup effect sizes and their corresponding 95% confidence intervals (CIs).

Publication bias assessment was performed with Begg’s funnel plot asymmetry test. The statistical analyses were performed with Stata software (Stata Corporation, Texas, USA) or with the Cochrane Review Manager software.

## 3 Results

### 3.1 Characteristics of the included studies

**Figure 1:**
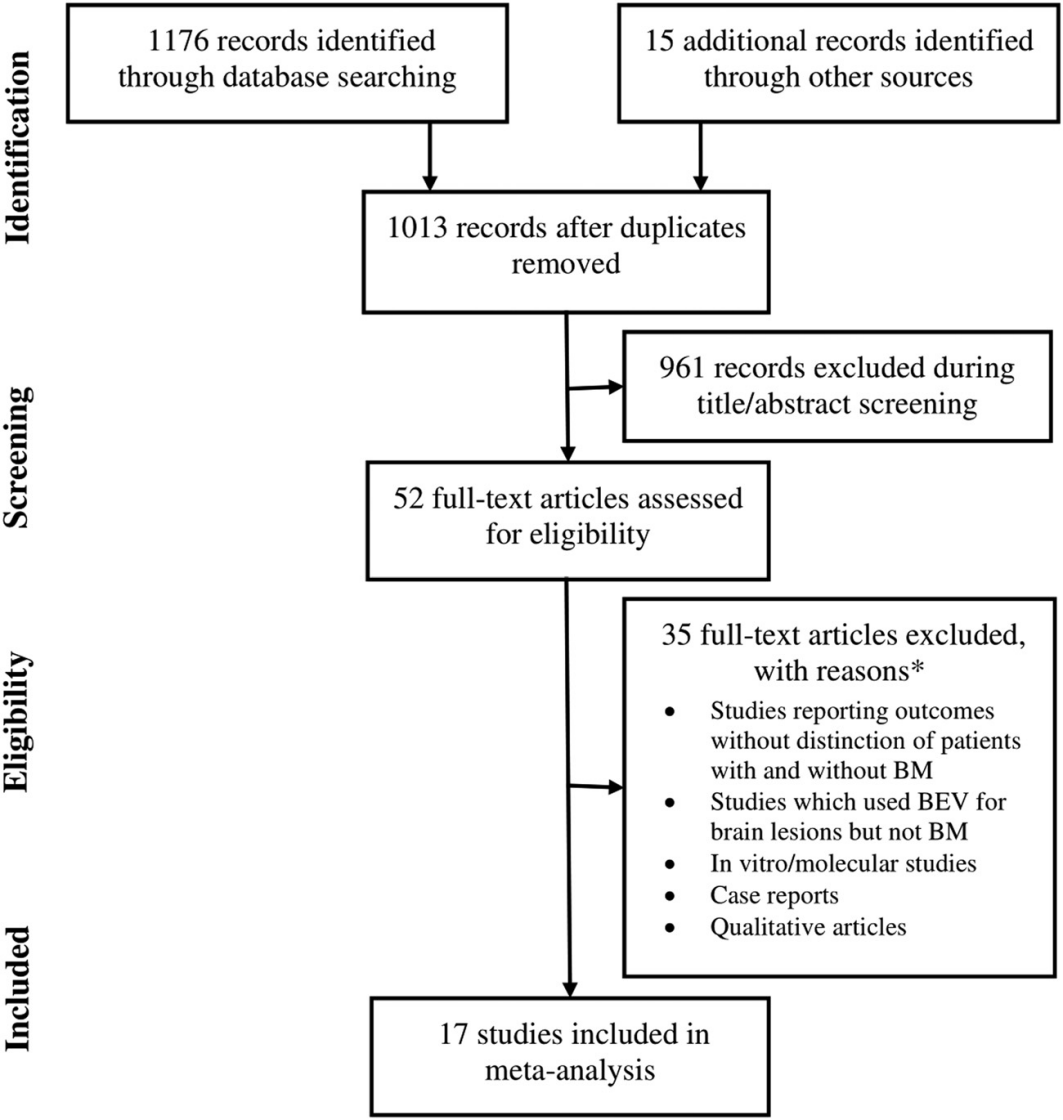
A flowchart of the study screening and selection process.

Seventeen studies [16–32] were selected based on the eligibility criteria (Figure 1). Fifteen studies had 7,212 NSCLC patients with BM at baseline, of which 1,548 were treated with BEV in combination with one or more therapies including chemotherapy, radiotherapy, stereotactic radiosurgery, and TKIs, and 5,664 patients served as controls who were treated with one or more of the above-mentioned therapies without BEV. Two studies recruited patients without BM at baseline (806 BEV-treated and 396 control patients) and followed them for the incidence of BM through follow-up. No significant publication bias was detected by Begg’s test (*P* = 0.245; Figure S1).

Important characteristics of the included studies are presented in Table S1, and study design and treatment features are presented in Table S2. Adenocarcinoma was predominant histological subtype of NSCLC, which was found in 87% [95% CI: 81, 92] of the patients, whereas large cell carcinoma was found in 10% [95% CI: 4, 17] of the patients. Most of the patients had an Eastern Ontario Oncology Group performance status of 0 or 1 (93% [95% CI: 84, 98]). The percentage of smokers (current or past) was 52% [95% CI: 38, 65]. In general, the quality of the included studies was above moderate. An assessment with New Castle–Ottawa scales is presented in Table S3.

### 3.2 Prevention of BM incidence

**Figure 2:**
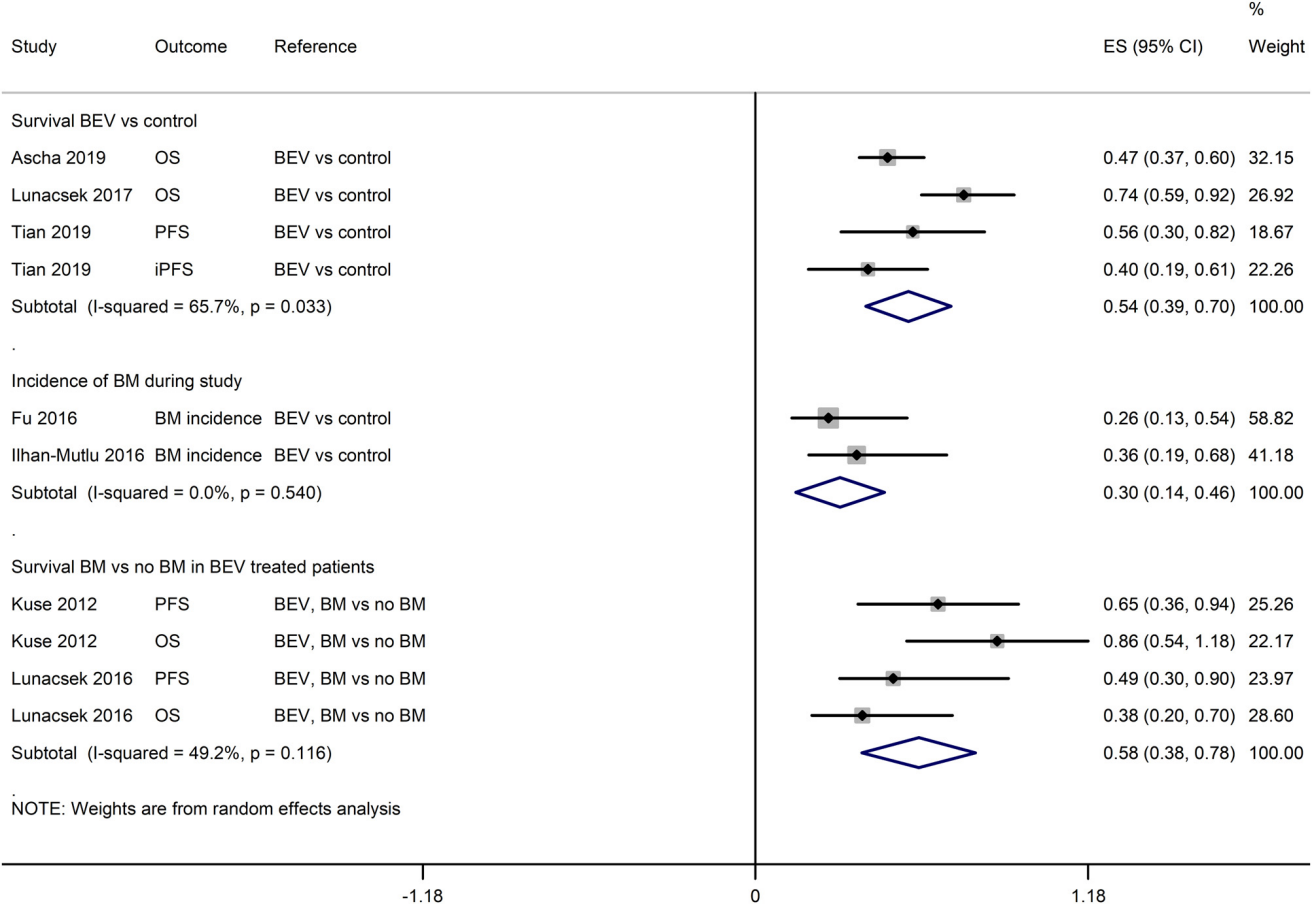
A forest graph showing the pooled HRs (effect size and 95% CI) regarding the incidence of new BM or survival to show BEV efficacy in preventing BM and survival outcomes.

In two studies that recruited NSCLC patients without BM at baseline, in comparison with chemotherapy alone, BEV treatment with chemotherapy was associated with low BM incidence in the NSCLC patients without BM at the start of study (HR: 0.30 [95% CI: 0.14, 0.46]; Figure 2).

### 3.3 Response rates

**Figure 3:**
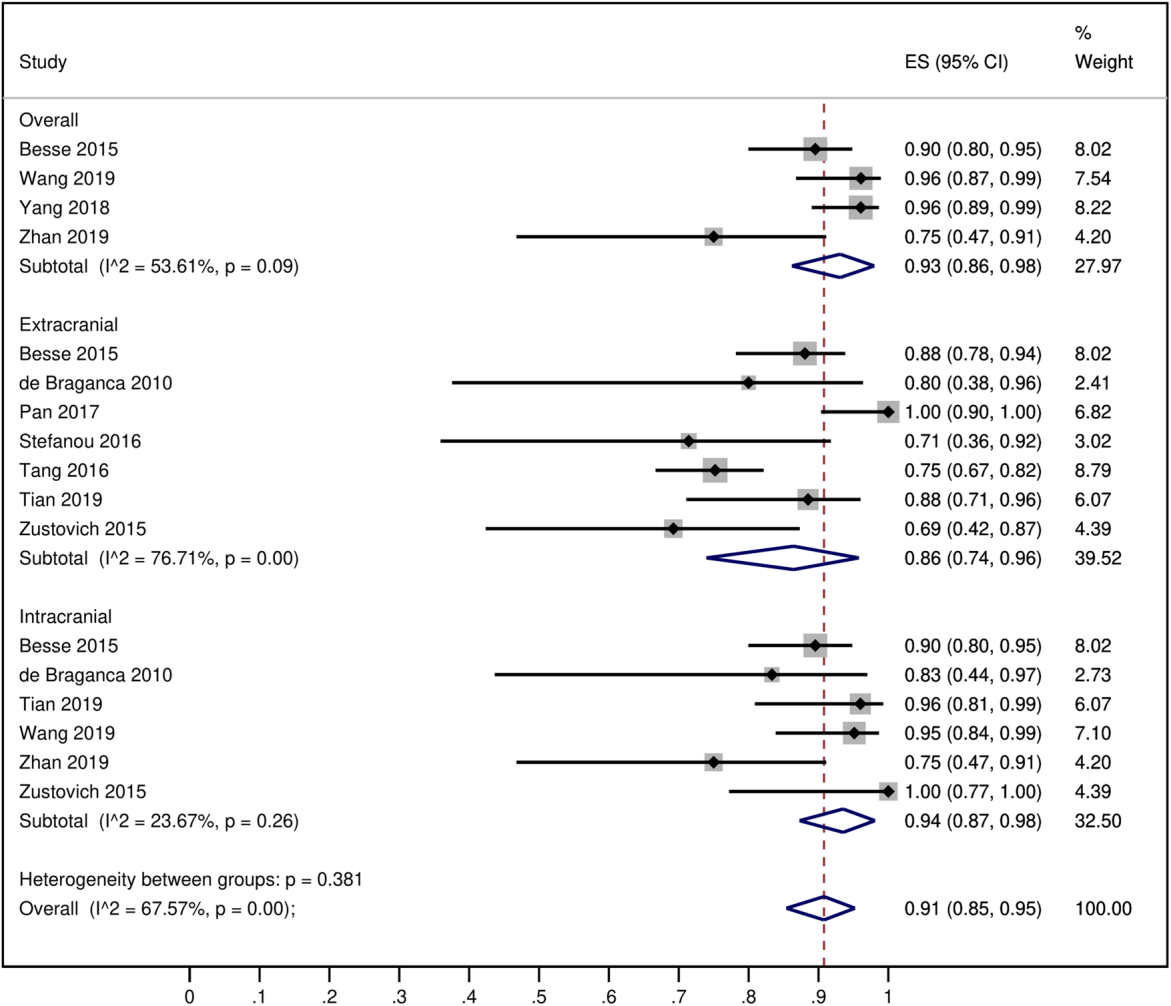
A forest graph showing the pooled DCRs of the BEV-treated NSCLC patients with the intracranial and extracranial subgroups. Subgroup “overall” represents the DCR of studies that reported DCR rates without distinguishing intracranial and extracranial DCRs.

**Figure 4:**
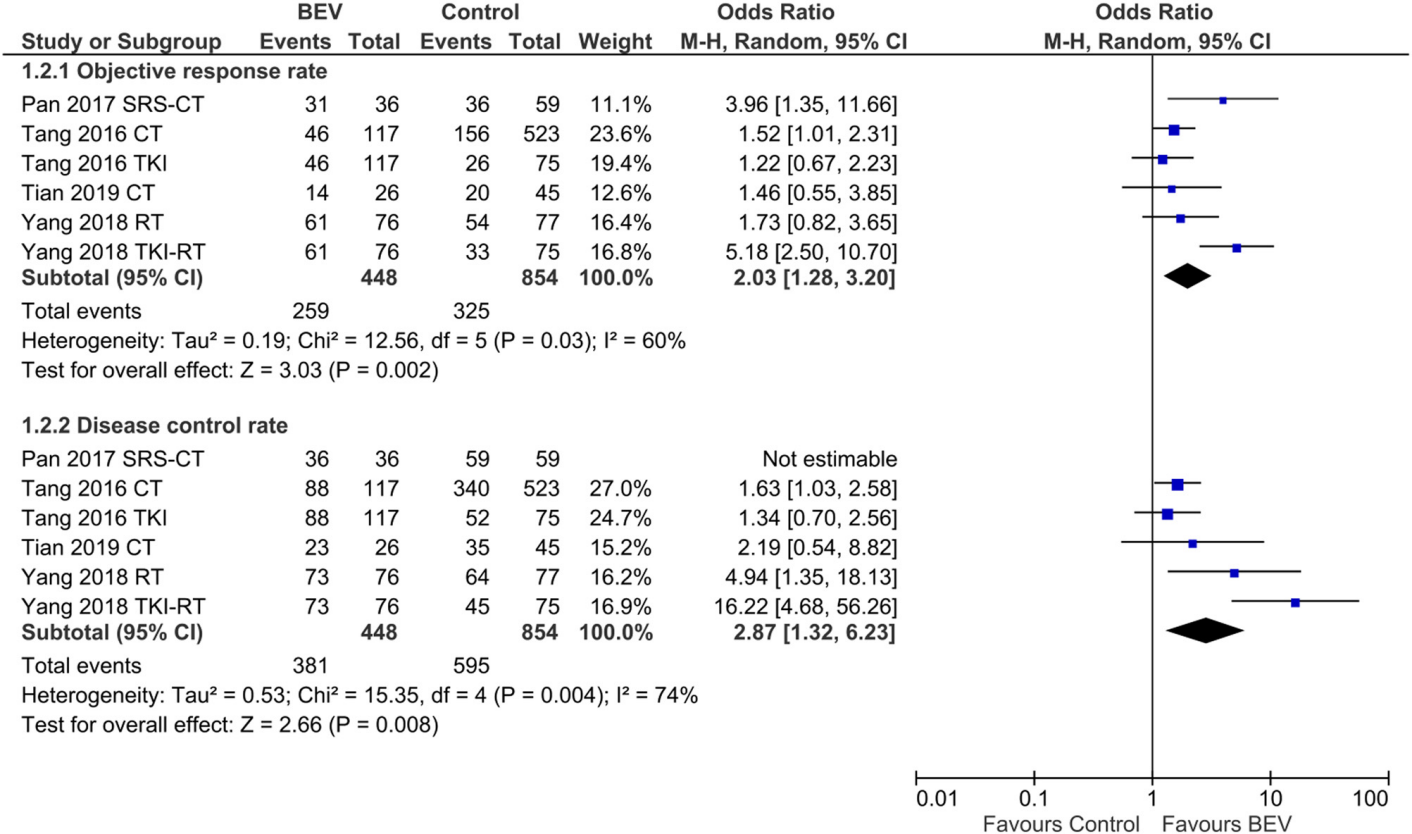
A forest graph showing the outcomes of the meta-analysis of the ORs between the BEV-treated and control patients in the ORR and DCR. Abbreviations in study identities: CT, chemotherapy; RT, radiotherapy; SRS, stereotactic radiosurgery; TKI, tyrosine kinase inhibitor.

In a pooled analysis, the overall DCR in the NSCLC patients with BM was 91% [95% CI: 85, 95]. However, the intracranial DCR was higher (94% [95% CI: 87, 98] than the extracranial DCR (86% [95% CI: 74, 96]) (Figure 3). In the NSCLC patients with BM, the ORR and DCR of the BEV-treated regimen were significantly better than those of the control treatments (OR: 2.03 [1.28, 3.20]; *P* = 0.002 for the ORR; and OR: 2.87 [1.32, 6.23]; *P* = 0.008 for the DCR; Figure 4). Correspondingly, the intracranial versus extracranial response rates of BEV-treated NSCLC patients with BM were 6% [95% CI: 0, 17] versus 0% [95% CI: 0, 4] for complete remission, 42% [95% CI: 33, 51] versus 47% [95% CI: 26, 68] for partial remission, 35% [95% CI: 26, 44] versus 28% [95% CI: 17, 40] for stable disease, and 5% [95% CI: 0, 13] versus 12% [95% CI: 3, 26] for progressive disease (Figures S2–S5).

### 3.4 Survival

**Figure 5:**
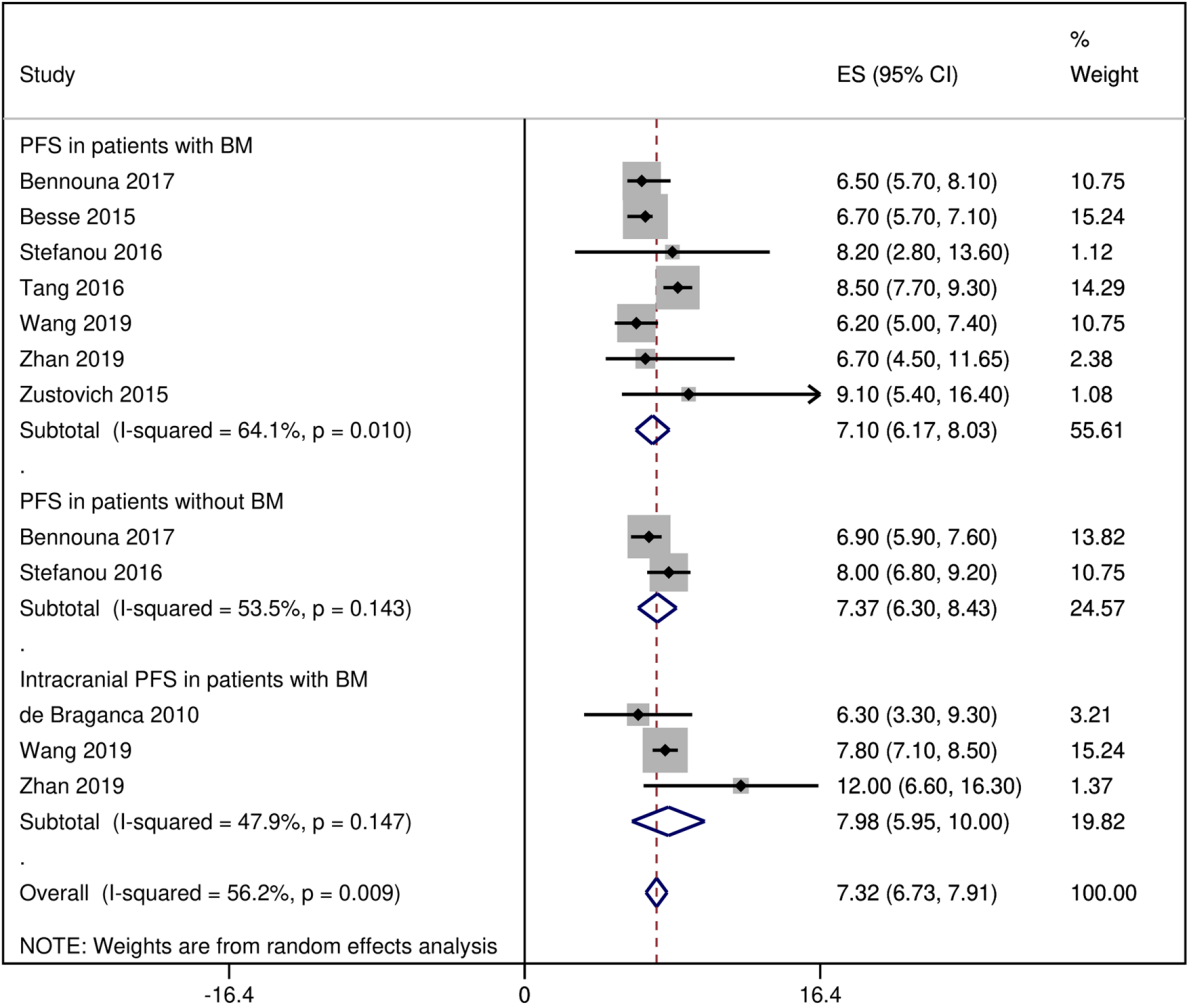
A forest graph showing the pooled overall and subgroup PFS rates of BEV-treated patients.

The PFS of the BEV-treated NSCLC patients with and without BM was 7.10 months [95% CI: 6.17, 8.03] and 7.37 months [95% CI: 6.30, 8.43], respectively, whereas the intracranial PFS of the BEV-treated NSCLC patients with BM was 7.98 months [95% CI: 5.95, 10.0] (Figure 5). The OS of the BEV-treated NSCLC patients with and without BM was 13.49 months [95% CI: 11.35, 15.62] and 12.49 months [95% CI: 10.22, 14.76], respectively (Figure S6). The 1-year OS in the NSCLC patients with BM was 66% [95% CI: 56, 74] (Figure S7). The pooled analyses of HRs reported by the individual studies indicated that the BEV treatment was associated with better survival in comparison with the control treatments in the NSCLC patients with BM (HR: 0.54 [95% CI: 0.39, 0.70]). Moreover, the BEV treatment was also associated with better survival in NSCLC patients with BM in comparison with NSCLC patients without BM (HR: 0.58 [95% CI: 0.38, 0.78]; Figure 2).

### 3.5 CNS bleeding

A meta-analysis of the studies that reported bleeding/hemorrhage in the CNS estimated the incidence at 1% [0, 3] with the BEV treatment as combinational regimen in the NSCLC patients with BM (Figure S8).

## 4 Discussion

This meta-analysis found that BEV treatment was associated with better efficacy in NSCLC patients with BM in comparison with control therapies; BEV efficacy was also better in patients with BM than in patients without BM; and BEV has preventive potentials for new BM incidence during the study follow-up. The incidence of intracranial hemorrhage was low (1%) in this population of NSCLC patients.

BM is one of the common complications of advanced NSCLC as more than 50% of NSCLC patients eventually develop BM [33]. BEV obstructs the VEGF pathway and has been shown to inhibit the growth of tumor cells by promoting tumor vascular degradation and normalizing existing tumor blood vessels [34,35]. Clinically, BEV-based treatments have become new therapeutic options for NSCLC patients with BM. However, only a few prospective studies have been made on BM from NSCLC, and debate continues about whether patients with BM can benefit from a BEV-based therapy.

The results of this meta-analysis show that as a first-line or maintenance therapy, BEV reduced the risk of a new incidence of BM from NSCLC by approximately 70% compared with chemotherapy or placebo. Blood circulation is a major path for tumor metastasis. As a VEGF inhibitor, BEV inhibits the growth of human tumor xenografts [36,37] and normalizes tumor blood vessels [35]. Therefore, we speculate that the possible mechanism of action of BEV is to prevent tumor cells from entering the blood vessels, which then reduces the incidence of BM.

BEV may normalize tumor blood vessels, which may result in a more efficient delivery and action of chemotherapies [35]. Pan et al. have reported that stereotactic radiotherapy combined with BEV to treat BM from pulmonary adenocarcinoma can achieve higher near-term tumor remission and perilesional edema control rates [24]. Furthermore, a study on mice showed that BEV not only inhibited angiogenesis but also enhanced the cytotoxicity of cisplatin and promoted apoptosis of tumor cells as a chemosensitizer [38]. It has also been found that low-dose BEV can also be used to treat brain edema due to radiotherapy [39].

Generally, the side effects of BEV therapy include hemorrhage/bleeding, wound healing complications, gastrointestinal perforation, arterial thromboembolism, congestive heart failure, hypertension, proteinuria/nephrotic syndrome, infusion-related hypersensitivity reactions, and reversible posterior leukoencephalopathy syndrome [40,41]. A meta-analysis of eight studies found that BEV treatment was not associated with a significant increase in intracerebral hemorrhage in cancer patients with BM [13]. In this study, the CNS bleeding rate with BEV treatment in NSCLC patients was 1%, which suggests that the risk of intracerebral hemorrhage is low with BEV treatment.

However, bleeding potential of BEV should not be overlooked altogether, as some studies have shown considerable bleeding events with BEV treatment. In a phase IV trial (SAiL) of the 2,212 non-squamous NSCLC patients treated with BEV, 38.2% had a bleeding event, of which 87% were resolved but 10% led to BEV discontinuation. The incidence of grade ≥3 pulmonary hemorrhage and intracerebral hemorrhage was 0.7% and 0.1%, respectively, in a previous study [42]. A meta-analysis also found that BEV treatment was associated with an increased risk of fatal pulmonary hemorrhage (relative risk 5.65 [95% CI: 1.26, 25.26]) in patients with lung cancer [12].

The meta-analysis of several indices reflecting the efficacy of BEV in the NSCLC patients with BM is the major strength of this study. Moreover, a reliable estimate of the incidence of CNS hemorrhage in the NSCLC patients with BM is also reported for the first time herein. However, some limitations of this study should also be noted: (1) no studies could be found from the literature to evaluate BEV versus other therapies with regard to survival, so a comparative account with regard to survival could not be made; (2) because of the observational design of the included studies, several types of biases could affect the outcomes; (3) in the pooled analyses, the subgroups were not balanced and hence *ad hoc* outcomes depending on future studies should be considered; and (4) the course of treatment was not uniform in all the studies. Therefore, a large, multicenter, randomized controlled trial is needed to verify further the safety and efficacy of BEV in the treatment of NSCLC patients with BM.

## 5 Conclusion

Our meta-analysis has shown that in NSCLC patients with BM, the ORR and DCR were significantly better with BEV-treatment than with contemporary therapies. The DCR and PFS of the BEV-treated patients were better for intracranial than for extracranial disease. Based on data from two studies, it was found that BEV reduced the risk of new BM incidence by 70%, which shows that it may have preventive potentials for BM. The risk of intracranial hemorrhage with BEV treatment was low.

## Abbreviations

AEs: adverse events
BEV: bevacizumab
BM: brain metastasis
CNS: central nervous system
CNKI: China National Knowledge Infrastructure
DCR: disease control rate
HR: hazard ratio
NSCLC: non-small cell lung cancer
OR: odds ratio
ORR: objective response rate
OS: overall survival
PFS: progression-free survival
TKI: tyrosine kinase inhibitor
VEGF: vascular endothelial growth factor
